# Food Provisioning and Parental Status in Songbirds: Can Occupancy Models Be Used to Estimate Nesting Performance?

**DOI:** 10.1371/journal.pone.0101765

**Published:** 2014-07-07

**Authors:** Aude Catherine Corbani, Marie-Hélène Hachey, André Desrochers

**Affiliations:** Centre d'étude de la forêt, Université Laval, Québec, Québec, Canada; Utrecht University, Netherlands

## Abstract

Indirect methods to estimate parental status, such as the observation of parental provisioning, have been problematic due to potential biases associated with imperfect detection. We developed a method to evaluate parental status based on a novel combination of parental provisioning observations and hierarchical modeling. In the summers of 2009 to 2011, we surveyed 393 sites, each on three to four consecutive days at Forêt Montmorency, Québec, Canada. We assessed parental status of 2331 adult songbirds based on parental food provisioning. To account for imperfect detection of parental status, we applied MacKenzie et al.'s (2002) two-state hierarchical model to obtain unbiased estimates of the proportion of sites with successfully nesting birds, and the proportion of adults with offspring. To obtain an independent evaluation of detection probability, we monitored 16 active nests in 2010 and conducted parental provisioning observations away from them. The probability of detecting food provisioning was 0.31 when using nest monitoring, a value within the 0.11 to 0.38 range that was estimated by two-state models. The proportion of adults or sites with broods approached 0.90 and varied depending on date during the sampling season and year, exemplifying the role of eastern boreal forests as highly productive nesting grounds for songbirds. This study offers a simple and effective sampling design for studying avian reproductive performance that could be implemented in national surveys such as breeding bird atlases.

## Introduction

Estimating reproductive performance is central to the understanding of population dynamics. In the case of birds, nesting success is typically measured through nest monitoring [1]. Nest monitoring delivers a direct and reliable estimation of parameters such as nest survival [2], and the presence of nestlings [3] and fledglings [4]. Some studies have monitored a large number of nests [3], [5], sometimes for a large sampling area and a high number of bird species [5], [6]. However, nest monitoring necessitates the use of cameras or sensors, or that nests be visited frequently by field biologists [7]–[9]. It is usually restricted to small areas [1], [10], [11], and to one or a small number of target species at a time, generally over small and intensively studied plots [11]. Moreover, despite their labor-intensiveness, nest monitoring studies may be subject to bias in the estimation of nesting success such as errors in nest fate estimation or partial predation [12].

Indirect methods can be effective for studying nesting success at the level of entire bird communities. They are often based on field observations of parental behavior, such as food provisioning, to determine the reproductive status [13]–[17]. However, these indirect methods suffer from imperfect detection because parental behavior is observable only part of the time. Moreover, parental behavior may vary according to sampling conditions. Food provisioning is dependent on prey availability [18], [19], and is known to fluctuate depending on time of day, with feeding activity generally concentrated in the early morning and evening [20]–[22]. Feeding frequency increases with ambient temperature [18], and high precipitations may limit food delivery [23], [24]. Thus to date, methods that are based on food provisioning have only yielded negatively biased indices of parental status and in turn, nesting success [10].

Specific modeling approaches have been developed for binomial observations with imperfect detection. MacKenzie et al. [25] and MacKenzie et al. [26] proposed a series of statistical tools to incorporate imperfect detection of occupancy within estimates, i.e. presence or absence of a target species from sites during the sampling period. They proposed a sampling protocol wherein repeated observations over time are made at several locations to resolve the ambiguity between genuine species absence and non-detection when species are not observed. Herein lies the strength of such a method: it accounts for imperfect detection and prevents underestimation of species occurrence in the studied area. Occupancy models potentially can be applied to other dichotomous states with imperfect detection, such as parental status, which is defined here as having offspring (nestlings or fledglings) or not. In principle, those two-state models can not only help assess the overall reproductive performance of avian populations, but also determine its variation through space and time, both of which are known to be important [27], [28]. Furthermore, two-state models can incorporate variation in the detection of food provisioning.

Here, we propose and evaluate an indirect method to assess parental status for individuals from multiple species of boreal forest songbirds. We used a novel combination of repeated observations of parental provisioning and two-state occupancy modeling [25], [26]. We applied this novel method to a boreal forest landscape at two different levels. First, we applied our method at the site level, with all individuals pooled. Site-level analysis allows an assessment of hotspots of parental activity in time and space and may be used to compare the phenology of nesting among different years. Second, we applied our method at the individual pair level to assess parental status on a per capita basis, thereby accounting for variation in population density. We modeled parental status, i.e. the proportion of sites or pairs with offspring (nestlings or fledglings) given by food provisioning observations, as a function of date and year, and detection of parental status as a function of sampling conditions (weather and time of day). We compared our parental status detection probabilities to estimates that were obtained by direct observations of food provisioning by adults with known active nests. The method proposed here is a potentially simple and efficient tool for assessing parental status, and could be implemented in national surveys such as breeding bird atlases.

## Materials and Methods

### Study area

The study was conducted at the Forêt Montmorency, a research forest that is managed for timber and which is located 70 km north of Québec City (47°20′N, 71°07′W). The landscape is hilly, with elevations ranging from 600 to 1100 m [29]. Mean annual temperature is 0.3°C, and mean annual precipitation is 1589 mm, with 40% falling as snow [30]. During the three summers of this study, mean ambient air temperature was similar to the long-term average (13.5°C), but precipitation was unusually scarce, notably in 2010 [30]. Coniferous forests covered 52% of the study area and were mostly mature (61- to 80-years-old). Mixed forests represented 34% of total cover and were mostly young (21- to 60-years-old). The remaining area had no forest cover and corresponded mainly to clearcuts, windthrows, and outbreaks of spruce budworm (*Choristoneura fumiferana)* [31]. Mature forest stands were dominated by balsam fir *Abies balsamea* (L.) Miller, accompanied by black spruce *Picea mariana* (Mill.) BSP, white or paper birch *Betula papyrifera* Marshall, and white spruce *Picea glauca* (Moench) Voss [32].

### Sampling parental provisioning

**Figure 1:**
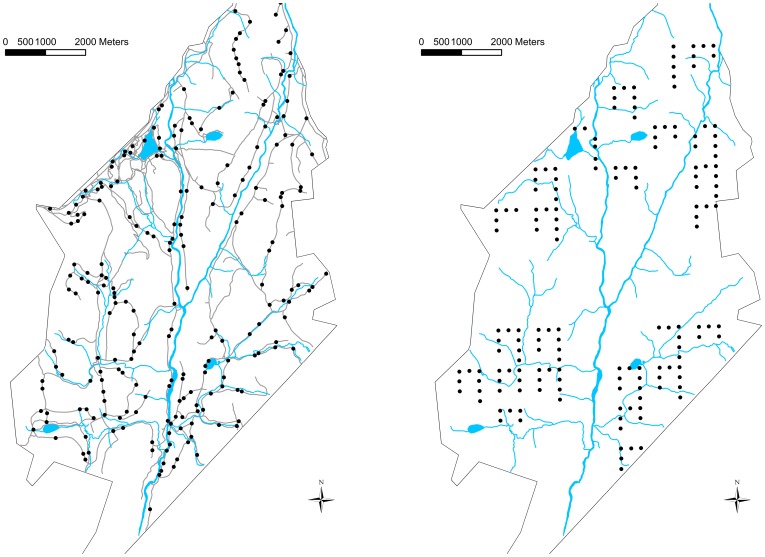
Sampling site locations at Forêt Montmorency (Québec, Canada). Sampling site locations on roads and trails (left) and systematic transects (right) at Forêt Montmorency (Québec, Canada) between 2009 and 2011. Blue lines and areas are rivers and lakes, while gray lines are unpaved forestry roads and trails.

We evaluated parental provisioning from 18 songbird species (Table 1) in 224 sites sampled from a set of random points that were placed >200 m apart along unpaved forestry roads and trails, and beyond forestry roads in 168 sites that were placed 250 m apart along systematic straight-line transects dispersed over the entire study area (Figure 1). None of the sites was visited in more than one year. We made three (2009) or four (2010–11) visits (sampling occasions) at each site, between 0500 and 1000 EDT, starting 28 May and ending 21 July, during days with no or little rain and no strong wind (<3 on Beaufort scale). The first observations of food provisioning were made on 4 June, 29 May and 15 June in 2009, 2010 and 2011, respectively. The last ones were made on 21 July in 2009 and 12 July in 2010 and 2011. We were able to complete all sampling occasions on consecutive days 46% of the time. In other cases, sampling was completed within 7 days. Two observers rotated every other day to survey sites. On each sampling occasion, we used a playback of Black-capped Chickadee (*Poecile atricapillus*) mobbing calls to draw birds in, to facilitate sighting and, in turn, to detect parental status (food provisioning: [15]). We played the mobbing calls with a five-watt amplifier that was connected to a digital audio player for a continuous duration of 15 min on each sampling occasion. We assume that birds responded to mobbing calls irrespective of nesting status because mobbing calls locally elicit strong responses even outside the nesting season [33], [34], and the number of birds that were attracted to mobbing calls at a given site did not vary according to date in the sampling period, despite strong differences in the proportion of birds provisioning their young (André Desrochers *unpublished data*).

**Table 1:** Total number of observed adults for each species and number of cases where parental provisioning was observed during the breeding season from 2009 to 2011 at Forêt Montmorency (Québec, Canada).

Common name	Scientific name	Number of site-species combinations	Number of times with adults seen carrying food
Yellow-rumped Warbler	*Setophaga coronata*	257	14
Red-breasted Nuthatch	*Sitta canadensis*	232	18
Magnolia Warbler	*Setophaga magnolia*	208	20
Black-throated Green Warbler	*Setophaga virens*	181	11
Ruby-crowned Kinglet	*Regulus calendula*	165	16
Philadelphia Vireo	*Vireo philadelphicus*	147	6
Bay-breasted Warbler	*Setophaga castanea*	146	18
Blackpoll Warbler	*Setophaga striata*	140	15
American Redstart	*Setophaga ruticilla*	132	22
Golden-crowned Kinglet	*Regulus satrapa*	131	21
Blue-headed Vireo	*Vireo solitarius*	106	6
White-throated Sparrow	*Zonotrichia albicollis*	95	7
Dark-eyed Junco	*Junco hyemalis*	93	9
Boreal Chickadee	*Poecile hudsonica*	86	7
Nashville Warbler	*Oreothlypis ruficapilla*	78	6
Swainson's Thrush	*Catharus ustulatus*	63	9
Least Flycatcher	*Empidonax minimus*	38	1
American Robin	*Turdus migratorius*	33	3
Total		2331	209

### Application of two-state models to parental status

The application of MacKenzie et al.'s [25], [26] likelihood-based method with two states necessitated an adjustment for parental status. This method was first developed for estimating the proportion of sites that were occupied by individuals or species when detection is imperfect. Here, we were interested not in species occupancy, but in parental status. We estimated the proportion of sites where there was at least one adult provisioning young, given occupancy by at least one species (Ψ_site_), together with the conditional probability that parental status is detected at each site at a given time, given the presence of adults with offspring (*p_site_*). Thus, for each visit at each site, we considered two outcomes, i.e. at least one adult carrying food regardless of species, vs. no adults carrying food given at least one adult was observed.

In a second analysis, we were interested in determining parental status at the individual pair level. We estimated the proportion of individual pairs provisioning young, given occupancy (Ψ_pair_), and the conditional probability that parental status is detected for each pair at a given time, given the presence of adults with offspring (*p_pair_*). We initiated a food provisioning detection history for each pair that was observed at a given site. For each site visit, we considered two outcomes, i.e. at least one member of the pair carrying food, or member(s) of the pair observed but not carrying food. To deal with cases where we made visual contact with more than one bird of the same species and sex, we constructed two datasets, one that classified pairs as provisioning (at least one adult of the species carried food vs. no adults of the species carrying food), and in the other dataset, we deleted the observational history, since we were unable to assign those birds to a specific pair. Consequently, we ran models twice for the analyses at the pair level. If no individual of the species was seen at one visit, a missing data point was recorded since parental activity was not sampled for that species. Furthermore, omitting those cases avoided the confounding of imperfect detection of parental status and imperfect detection of the birds themselves.

### Assumptions of two-state models

Models and analytic tools that are used here are an extension of traditional closed-population capture-recapture theory [35], [36]. Several assumptions need to be met and are adaptable in principle to the study of parental status. First, in all analyses we assume that all birds carrying food had either nestlings or fledglings. This assumption is borne out by the fact that the only other reason for carrying food is courtship feeding, which is seldom observed in passerines except for chickadees and nuthatches ([37] and pers. obs.) and appears mostly very early in the nesting season.

A second assumption in using the common occupancy model is that parental status did not change at given sites during the sampling period (closure assumption; [38]). The closure assumption took different meanings here depending on the level of analysis. When estimating parental status at the individual pair level, the closure assumption required that parental status of given pairs did not change during consecutive days of sampling at a given site (details above). Given that the entire nesting cycle lasts ∼26 days from egg-laying to fledging in the species that were studied [39], not to mention days or weeks of fledgling care, and given that we conducted multiple visits within only a few days, thereby preventing renesting attempts, we assume that changes in nesting state that were due to hatching during the course of a visual detection history have occurred rarely. However, changes in parental status due to nest failure in the course of a visual capture history may occur more frequently, thereby compromising the closure assumption. To address this concern, we compared the frequency of cases where parental status was detected initially but not on later occasions (“1-0” or “1-•-0” sequences in visual capture histories), to the converse cases (“0–1” or “0-•-1”sequences). A greater frequency of “1–0” sequences could be interpreted as evidence of lack of closure due to nest failure. When estimating parental status at the site level, the closure assumption is relaxed, requiring only that the presence of nestlings *of any species* did not change over consecutive sampling days at a given site. Failure to meet the closure assumption in the latter case would occur only if 1) none of the breeding birds at the site initially had nestlings but at least one of those birds hatched eggs during the sampling period at the site or 2) all of the nestlings or fledglings that were initially present at the site died before the end of the sampling period at the site. We assume that both of those two cases were not sufficiently frequent to bias estimates substantially.

In individual-pair analyses, a third assumption is that pairs observed at a given site did not change between visits on consecutive days, i.e. individuals of a pair were correctly identified. We considered this assumption as reasonable because during the breeding period, individuals of the same species defend territories that do not overlap, and the spatial response to mobbing calls is restricted by territory boundaries [40]. Nevertheless, we examined this assumption by monitoring color-banded individuals (details below) over consecutive days at 15 fixed locations (mean nearest-neighbor  = 112 m), using the same mobbing call playback procedure as described above, to measure the proportion of times birds of a given species that were drawn to a site on consecutive days were the same individuals. Last, we addressed statistical independence among sites by measuring the spatial autocorrelation of site-specific estimates of parental status.

### Software requirement and covariates

We implemented single season, two-state models with the Unmarked package 0.9–7 in R Version 2.15.3 [41], [42]. This package enabled us to estimate the proportion of sites or pairs with offspring, as well as the detection probability of food provisioning, given parenthood, as a function of several covariates which we had deliberately limited in number. Limiting the number of covariates let us portray a simple picture of parental status for the entire songbird community over a forest landscape. The closure assumption was required only during the three or four consecutive visits; thus, we allowed longer-term changes in parental status by modeling the effect of Julian day (mean number of days since 1 January (of the visits to) at a given site) on parental status. Also, we considered year and its interaction with Julian day as regressors. Sampling time of day and meteorological conditions, i.e. hourly ambient temperature (°C) and hourly rainfall (mm), were modeled because of their potential influence on parental provisioning and therefore, on detection probability. Weather data were obtained from the weather station that was located within the study area (Forêt Montmorency; [30]). Data sets are available in Table S2 and covariates were standardized during the modeling process. With the null model and the full one (containing all covariates), we compared six models for each studied level (i.e. site- and pair-level) of parental status based on the Akaike information criterion (AIC) [43] and when necessary used multi-model inference based on model averaging [43]–[45]. The goodness-of-fit of the best model was tested with 1000 parametric bootstraps [41], [46].

### Validation of two-state models

Estimates of detection probability of parenthood needed evaluation based on direct and independent assessment of parental behavior. For this purpose, we observed food provisioning by adults with known active nests. Between 25 May and 7 June 2010, we mist netted 138 individuals of 25 species (Table S1) in a 1.1 km^2^ core section of the study area, individually marked them with two colored plastic and one aluminum numbered bands, and searched for their nests. Sixteen nests of 8 species (Table 2) were found and were visited daily or every two days to determine hatching date. When the exact hatching date was unknown, we subsequently inferred it from the fledging date, based on the reported number of days for incubation and nestling stage for each species [39]. Nests that were higher than 2 m were checked with a mirror mounted on a telescopic pole. After hatching, we conducted observations of food provisioning around nests by each parent at distances between 0 and 249 m from nests, without using mobbing call playbacks. We searched for adult birds away from each of the 16 nests that were found by following the recommendations of Martin and Geupel [1]. When found, color-banded focal individuals (*n* = 7) were subjected to instantaneous sampling [47] of parental provisioning, between 0500 and 1200 EDT. Focal birds were followed for a minimum of 15 s (digital watch) before data were collected. This delay was implemented to reduce possible discovery bias due to changes in behavior [48]. We recorded whether or not the focal bird was carrying food, together with the location of the bird by GPS. We recorded not more than one instantaneous parental provisioning sample per foraging round trip, to ensure independence among observations of parental provisioning. Twenty-five unbanded adults with known nests were also sampled, with one observer monitoring them while the other observer remained at the nest to confirm their identity in cases where other unbanded adults of the same species were present in the area.

**Table 2:** Total number of monitored nests and observations for each species and number of cases where parental provisioning was observed during the breeding season in 2010 at Forêt Montmorency (Québec, Canada).

Species	Scientific name	Number of nests	Total number of observations	Number of times with adults carrying food
White-throated Sparrow	*Zonotrichia albicollis*	5	67	23
American Redstart	*Setophaga ruticilla*	3	89	28
American Robin	*Turdus migratorius*	2	40	17
Boreal Chickadee	*Poecile hudsonica*	2	28	5
Chipping sparrow	*Spizella passerina*	1	44	13
Rusty Blackbird	*Euphagus carolinus*	1	42	7
Philadelphia Vireo	*Vireo philadelphicus*	1	17	7
Dark-eyed Junco	*Junco hyemalis*	1	4	2
Total		16	331	102

We modeled parental provisioning detection with a mixed-model logistic regression. We tested the influence of the following fixed effects on the detection of parental provisioning: time of day, ambient temperature, and hourly precipitation. We also included as fixed effects the relative age of the nestlings (between zero and one, one corresponding to the mean fledging age for the species; [39]), a factor that may influence feeding frequency ([49] and references therein), and the log-distance to the focal nest because of its potential effect on parental status [10], [50]. Distances between nests and provisioning observations were calculated within ArcGIS ([51]). We included nest ID within species (16 nests, 8 species) as random effects, reflecting the repeated parental provisioning observations made on each nest. There was no collinearity between effects of time of day and temperature (*r* = 0.3, variance inflation factor  = 1.3). All statistical analyses were performed using the lme4 package in R 2.15.3 [42].

## Results

### Proportions of sites with adults provisioning young

A high proportion of sites contained adults provisioning young at the end of the breeding season, and parental status was detected to a maximum of 58% among sites. We had a visual contact of 48% of the birds within the first 5 minutes, and 80% within the first ten minutes of the 15-min visits. We observed food provisioning at 123 of the 393 sites sampled. In 57% of the sites, we were unable to conduct surveys on consecutive days because of weather conditions (mean time between first and last visit: 4.6 days; maximum: 7 days). The model that performed best was the full one, i.e. the model containing all possible covariates for each parameter (Table 3). Its adjustment was verified with 1000 parametric bootstraps and we found that our best model fitted the data adequately (*P* = 0.4). The detection probability of parental status at given sites increased from the beginning (1 June) to the end of the field season (18 July), based on the best two-state model estimates (Table 4). Detection probabilities ranged from 0.07±0.14 (± standard error) to 0.58±0.08, depending on covariate values that were considered. After accounting for imperfect detection, the estimated proportion of sites with adults provisioning young varied from 0.01±0.01 early in the season to 0.85±0.11 at the end of the sampling season depending on the sampling year that was considered (Table 4).

**Table 3:** Ranked two-state models of parental status (Ψ_site_; according to Julian day and sampling year) and its detection probability (*p_site_*; according to sampling conditions) at site level (*n* = 393 sites).

Model structure		No. parameters†	ΔAIC	Akaike weight
Ψ_site_	*p_site_*			
Full	Full	15	0	0.98
Julian day × sampling year	Julian day × sampling year	12	8.03	0.02
Julian day	Julian day	4	18.44	0
Sampling year	Sampling year	6	53.87	0
Null	Null	2	65.86	0
Null	Sampling conditions‡	5	65.90	0

Julian day is the number of days since 1 January of the corresponding year.

AIC of the highest-ranking model: 999.17.

† Intercept parameters for Ψ and *p* were included in all models.

‡ Sampling conditions included time of day and sampling weather conditions as covariates of parental status detection probability.

**Table 4:** Estimated parameters (logit scale) and their standard error (SE) of parental status (Ψ_site_) and its detection probability (*p_site_*) for the best model (Ψ(Full), p(Full)) at site level.

Parameters	Ψ_site_				*p_site_*			
	Estimate	SE	*Z*	*P*(>|*z*|)	Estimate	SE	*Z*	*P*(>|*z*|)
Intercept	−0.619	0.450	−1.376	0.2	−0.260	0.553	−0.470	0.6
Julian day	2.270	0.745	3.049	0.002	0.249	0.500	0.498	0.6
Sampling year 1†	0.905	1.956	0.463	0.6	−0.2192	1.002	−0.219	0.8
Sampling year 2†	0.667	0.515	1.295	0.2	0.592	0.444	1.332	0.2
Julian day × sampling year 1†	−2.348	1.581	−1.485	0.1	0.447	0.885	0.505	0.6
Julian day × sampling year 2†	−1.658	0.816	−2.033	0.04	0.206	0.572	0.360	0.7
Time of day	-	-	-	-	−0.191	0.111	−1.721	0.08
Hourly ambient temperature	-	-	-	-	−0.067	0.027	−2.480	0.01
Hourly rainfall	-	-	-	-	−0.033	0.019	−1.762	0.08

Julian day is the number of days since 1 January of the corresponding year.

† Sampling year 1 corresponded to 2009 and sampling year 2 corresponded to 2010 (2011 is the sampling year of reference).

### Proportions of pairs provisioning young

**Figure 2:**
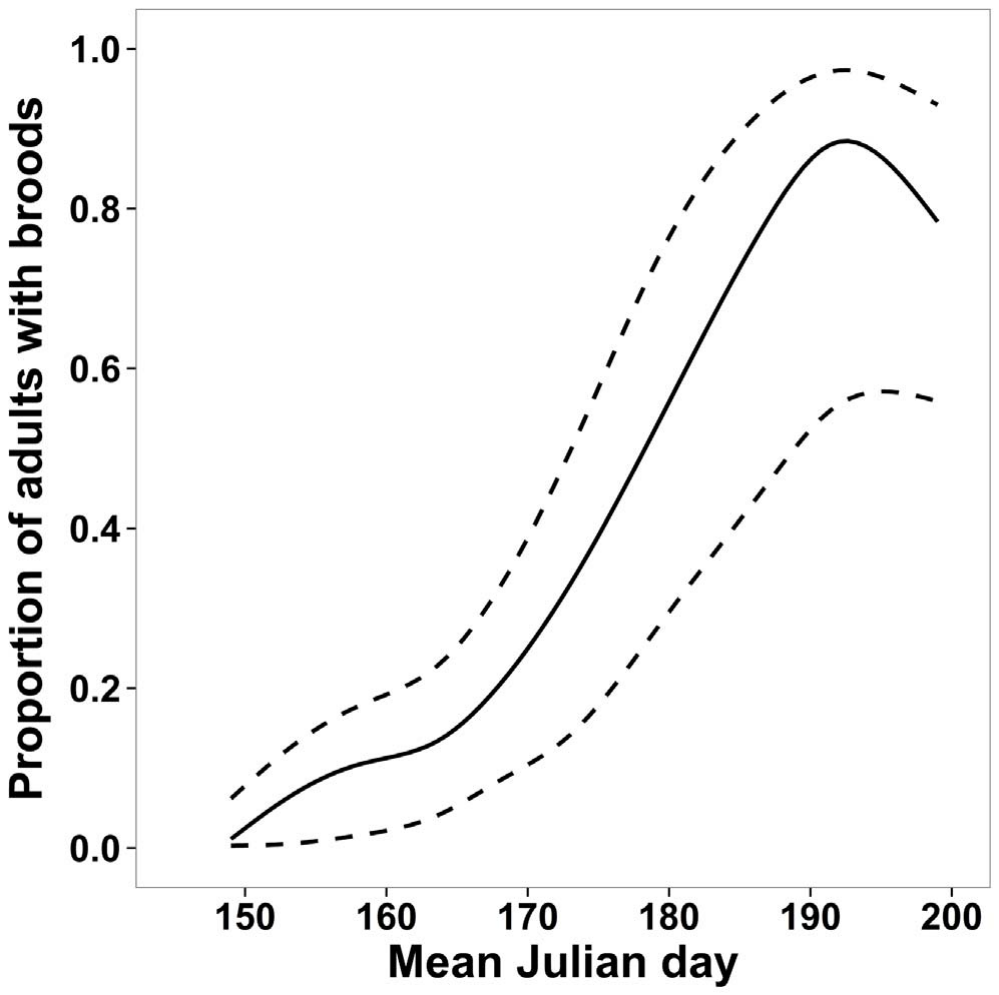
Estimated parental status at pair level. Model averaged estimated parental status (Ψ_pair_) at pair level as a function of mean Julian day (first observation Julian day: 28 May  =  day 148) at Forêt Montmorency (Québec, Canada). Dashed lines delimit the 95% confidence interval.

Most pairs provisioned young in late July, and detection probability of parental status for given pairs never exceeded 38%. The suppression of ambiguous cases, i.e. more than one individual of the same species and sex at the same site (*n* = 41), did not change results substantially. To maintain the entire dataset, we thus retained the approach including more than one individual of the same species and sex at the same site. During the three sampling seasons, we obtained 2331 food provisioning detection histories, i.e. we determined the parental status of 2331 adult songbirds. We observed food provisioning 209 times in 18 species (Table 1). Visual detection histories containing one, two, three and four observations occurred in 67%, 23%, 8%, and 2% of cases, respectively. No model performed better than the other competing models (AIC difference between first and subsequent models <4; Table 5); thus, we only used model averaging to assess the significance of each covariate. We verified the adjustment of our modeling on the first model based on its AIC with 1000 parametric bootstraps and we found that it fitted the data adequately (*P* = 0.3). Based on model-averaged estimates (Table 6), only mean Julian day of the visit affected parental status and its detection probabilities (95% confidence intervals excluded zero). The other covariates (i.e. sampling year, time of day and weather conditions) affected neither parental status nor detection probabilities. The detection probability of parental status decreased from the beginning (1 June) to the end of the field season (18 July). Detection probabilities ranged from 0.11±0.02 to 0.38±0.11, depending on the date that was considered. After accounting for imperfect detection, the estimated proportion of adults provisioning young increased sharply from 0.01±0.01 at the beginning of the field season to 0.90±0.08 at the end of the sampling season (Figure 2). Thus, results at the individual pair level were consistent with those at the site level, with a low detection probability of parental care with most adult songbirds rearing nestlings or fledglings by mid-July.

**Table 5:** Ranked two-state models of parental status (Ψ_pair_; according to Julian day and sampling year) and its detection probability (pair; according to sampling conditions) at pair level (*n* = 2331 adult songbirds).

Model structure		No. parameters†	ΔAIC	Akaike weight
Ψ_pair_	*p_pair_*			
Julian day	Julian day	4	0.00	0.59
Julian day × sampling year	Julian day × sampling year	12	1.26	0.31
Full	Full	15	3.61	0.10
Sampling year	Sampling year	6	102.61	0.00
Null	Null	2	110.96	0.00
Null	Sampling conditions‡	5	112.35	0.00

Julian day is the number of days since 1 January of the corresponding year.

AIC of the highest-ranking model: 1419.85.

† Intercept parameters for Ψ and *p* were included in all models.

‡ Sampling conditions included time of day and sampling weather conditions as covariates of parental status detection probability.

**Table 6:** Model averaged estimates (MAE; logit scale) and their unconditional standard error (USE) of parental status (Ψ_pair_) and its detection probability (*p_pair_*) at pair level; confidence interval (CI) not overlapping zero indicated estimates that were significantly different from zero.

Parameters	Ψ_pair_				*p_pair_*			
	MAE	USE	<CI	>CI	MAE	USE	<CI	>CI
Intercept	−1.241	0.550	−2.320	−0.163	−1.255	0.515	−2.265	−0.245
Julian day	2.070	0.312	1.458	2.683	−0.452	0.169	−0.783	−0.120
Sampling year 1†	1.865	1.242	−0.570	4.300	−1.215	0.753	−2.690	0.261
Sampling year 2†	0.387	0.476	−0.547	1.321	0.280	0.470	−0.641	1.200
Hour	-	-	-	-	−0.083	0.090	−0.259	0.093
Temperature	-	-	-	-	−0.032	0.023	−0.078	0.014
Precipitations	-	-	-	-	−0.008	0.014	−0.036	0.020

Julian day is the number of days since 1 January of the corresponding year.

† Sampling year 1 corresponded to 2009 and sampling year 2 corresponded to 2010 (2011 is the sampling year of reference).

### Evaluation of two-state models assumptions

As presented in the Materials and Methods section, key assumptions had to be satisfied to use two-state models. Nest predation did not appear to violate the closure assumption of analyses at the individual pair level, since the number of “1–0” sequences was not significantly greater than that of “0–1” capture histories (58 vs. 55, one-tailed binomial test: *P* = 0.4). Thirty-three color-banded individuals were drawn to mobbing calls at 15 sites in the validation part of the study area, and there were only two cases with different individuals of the same species being observed at the same site on two different days. Furthermore, only 8% of surveys included more than one individual of the same species and sex, thus supporting the “same individual” assumption specific to individual pair analyses. The spatial autocorrelation of estimated probabilities of parental status detection was very small (Moran's *I*<0.05 at the analysis levels). Thus, none of the model assumptions that were presented in the Materials and Methods section appeared to have been violated.

### Validation of two-state models

Parental status detection probabilities were compared to estimates that had been obtained by direct observations of food provisioning by adults with known active nests. To obtain such estimates, food provisioning was studied for 16 nests of 8 species (Table 2), at distances ranging between 0.3 m to 249 m (mean  = 38 m) from their nests. Of 331 instantaneous sampling observations, birds were seen carrying food on 102 occasions. The modeled probability of (or proportion of time) transporting food was 0.31 (95% confidence limits: 0.25–0.37) and, therefore, within the range estimated by the best two-state model. Contrary to the two-state models, time of day was significantly associated with the proportion of time that adults spent carrying food (Table 7). The monitoring of birds with known nests was conducted between 0500 and 1200 EDT, whereas repeated visits for two-state models were based on a narrower range of times (0500 - 1000 EDT). When running both models strictly based on observations made between 0500 and 1000 EDT, time of the day still had a significant negative effect on detection probability (*P* = 0.007). There was a slight but significant negative effect of distance to nest on the probability of observing food provisioning (Table 7). Nestling age, temperature and precipitation were not related to the proportion of time that adults spent carrying food (Table 7).

**Table 7:** Relationship between probability of observing food provisioning, age of nestlings, time of the day, temperature, hourly precipitation and distance of parent to nest in 2010 at Forêt Montmorency (Québec, Canada); Pearson correlation coefficient between observed and fitted frequencies: *r* = 0.20 (*n* = 16 nests).

	Estimate	Std. error	*Z*	*P*
(Intercept)	2.49	1.06	2.34	0.02
Relative age	−0.41	0.70	−0.59	0.6
Time of day	−0.23	0.09	−2.64	0.008
Temperature	−0.01	0.03	−0.29	0.8
Precipitation	−0.96	0.93	−1.04	0.3
Log-distance to nest	−0.30	0.14	−2.18	0.03

## Discussion

Occupancy models for dichotomous variables such as parental status appear to be promising as a tool for evaluating reproductive performance of songbirds over entire communities and landscapes. By combining repeated observations of parental provisioning within a short time frame and hierarchical modeling, we were able to account for imperfect detection and obtained point estimates of parental status for an entire breeding bird community over a 66 km^2^ landscape. Three site visits of 15 minutes are not trivial in terms of the labor that is required, but they are far less labor-intensive than systematic nest searches. Our ability to find and monitor only 16 nests in an entire nesting season, despite the full-time work of two field ornithologists, exemplifies well the labor cost of direct nest monitoring, at least in a boreal forest such as the one where we conducted this study.

To be sure, several caveats will need to be addressed more extensively to ascertain the reliability of the method we propose here. As is the case with other uses of avian occupancy modeling, lack of closure in the state of the system being sampled is a potentially serious issue [38]. As noted in the Materials and Methods, the duration of the hatching period relative to the entire nesting cycle (from egg-laying to fledgling independence) is probably sufficiently short to avoid significant lack of closure in parental status. Even in the case where eggs hatched during the sampling period, feeding frequency typically remains low in first days after hatching [49], [52], [53], thus does not lead to an abrupt change in parental status. Furthermore, the similar frequencies of “1–0” and “0–1” sequences in visual capture histories lent further support to the closure assumption and suggested that nest predation was infrequent, as inferred from artificial nest studies that have been previously conducted in the same area (nest predators generally are present at <30% of stations and <20% of baits depredated in [17], [54]). To further reduce the possibility of violating the closure assumption, the time interval between consecutive visits to a site could be reduced, e.g. only two visits during consecutive days, two visits per day, or even divided into separate sampling intervals (e.g. each 15 min visit could be recorded as three 5-min counts). However, very short time intervals between visits involving the use of playbacks could create independence and bird habituation issues [55].

Although coarser than the pair-level analysis, the site-level analysis was more robust to assumptions. It did not require that the same individuals be observed from one visit to the other. Site-level assessment of parental activity may be sufficient in most cases, to inform on spatiotemporal variation of reproductive activity, both within- and among-years. Despite our validation with known nests, our pair-level analysis could be criticized because we had a small sample size for validation. Yet, variation in provisioning behavior proved sufficiently small within and among individuals to yield statistically-significant spatial and temporal patterns in food provisioning and thus, a useful benchmark for the statistical approach accounting for detection. Obviously, more empirical data would be welcome to further document variation in provisioning behavior and, thus, provide a better benchmark to further validate our method. Misidentification of individuals remained possible, due to potential presence of floaters [56] and extraterritorial forays by conspecifics [57], [58]. However, we almost always re-observed the same individual of a species at the same site on two different days as shown in the validation part of the study area. More significantly, estimates of food provisioning were similar at the site- and the pair-level, suggesting that the precautions we took in identifying and assigning individuals to a given pair from one visit to the next were sufficient and consistent.

The estimates of parental status detection broadly agreed between two-state modeling and intensive focal individual sampling. Adults provisioning young generally spent <30% of the time carrying food, highlighting the need to take imperfect detection into account when estimating parental status from parental activity. The use of occupancy models to estimate parental status appears robust and offers an elegant way to assess songbird reproductive performance. We interpret the greater variability in the estimates of parental status detection at the level of sites vs. pairs as due to lower sample size (393 vs. 2331 sampling histories). Also, similar estimates between two-state modeling and intensive focal individual sampling indicated that there was no evidence of bias due to the use of mobbing call playbacks in the two-state modeling approach. In any case, it is important to note that the use of mobbing call playbacks is a nonessential part of the two-state modeling approach; its goal was simply to increase the number of provisioning detection histories.

Parental status detection varied according to sampling conditions, i.e. time of the day and weather conditions (hourly rainfall and ambient temperature). By measuring these variations, we could assess and compare parameters of interest (here, parental status) even if sampling conditions changed. Variation in parental status detection according to different parameters (particularly those affecting parental status itself) demonstrates that it is essential to move beyond nesting success indices such as those proposed by Vickery et al. [13], which did not consider parental status detection, and to use hierarchical modeling as presented here to estimate reproductive performance of birds. Here, parental status detection varied during the sampling season, and according to time of the day among sites or pairs. Food provisioning may change with time during the season because of seasonal change in brood size [49], predation risk [59], or food availability that might constitute a determinant of bird breeding success [60]–[62]. It also may change during the same journey [21], with feeding activity generally concentrated in the early morning and evening [20], [22], [63]. Likewise, bird activity is dependent on temperatures [18], [64], and we showed that hourly ambient temperature acted negatively on detection at the site level. Contrary to sampling time and conditions, distance to nest cannot be incorporated into a two-state model approach based on parental activity because the nest location is usually unknown. Thus, any bias due to distance to nest could be seen a major limitation of this approach. Fortunately, based on the nest monitoring portion of this study, distance to nest only had a limited effect on the detection of parental status. Furthermore, it appeared reasonable to assume that, under most circumstances, distances of focal birds to their nest would be random with respect to the covariates under study.

Even though we did not measure nesting success or brood fate *per se*, it can be inferred, in principle, from the proportion of sites with parents or the proportion of observed adults provisioning young at the sampling time [15]. In the case of a short (synchronous) nesting season, as was the case in the present study [39], the peak proportion of adults or sites with offspring informs us about nesting success; a maximum estimate of 90% of adults having broods suggests that nesting success is extremely high in our study area. However, it may prove difficult to infer nesting success with our method in ecosystems where nest predation is very high, because of important departures from the closure assumption.

Our estimates of parental status were consistent with the frequently held idea that boreal forests constitute major nurseries for North American birds [65] with a vast majority of adults tending broods in late summer. From a demographic perspective, the maximum estimates that were obtained from models incorporating a temporal component (here, Julian date) arguably provided the most useful information about nesting success. Yet, we need to reemphasize that our methodology does not provide an assessment of reproductive success in the usual way that this is defined. Instead, our approach provides point estimates of parental activity in time and space, which is indicative of reproductive success, but this is also valuable information in itself because of its high spatial and temporal resolution. A simulation study would be needed to quantify the link between point estimates of parental status such as in this study, and nesting success, which is usually defined as the proportion of nests leading to at least one fledgling. In locations with extended nesting seasons, a more complex modeling exercise would be required, for example by comparing different combinations of nesting asynchrony and daily nest survival probabilities.

## Conclusion

Over the last decade, several studies have expressed the need to deliver quantitative information on the productivity of entire communities of songbirds, over large geographical areas [10], [11], [66]–[68]. Yet, the best response offered to date is a very small number of studies on limited numbers of species (e.g. [3], [4], [6], [9]) or those yielding relative indices of nesting success [13], [15]–[17], [69]. The method proposed here is a potentially simple, effective and reliable tool for the assessment of parental status in a variety of environments. It therefore has some of the essential attributes for potential implementation in national surveys such as breeding bird atlases. It uses data that are frequently collected in songbird inventories. We are hopeful that it also will contribute to a better understanding of avian reproduction in various ecosystems, including the boreal forest.

## Supporting Information

Table S1 **Total number of mist-netted and banded adults for each species during the breeding season in 2010 at Forêt Montmorency (Québec, Canada).**
(DOC)

Table S2 **Observations made during the successive visits at site (** ***n*** **= 393) and pair (** ***n*** **= 2331) levels in 2009–2011, and corresponding site (mean Julian day of visits) and visit covariates (hourly ambient temperature and rainfall, time of day).** “1” indicates observation of food provisioning and “0” indicates adult(s) observed without food. All covariates were standardized during the modeling process.
(XLS)
