# Supplementary material for: Food Provisioning and Parental Status in Songbirds: Can Occupancy Models Be Used to Estimate Nesting Performance?
Source: PLoS One. 2014 Jul 7;9(7):e101765. doi: 10.1371/journal.pone.0101765 (PMC4084988; doi:10.1371/journal.pone.0101765)
Supplement: Table S1 — Total number of mist-netted and banded adults for each species during the breeding season in 2010 at Forêt Montmorency (Québec, Canada). (DOC) [file pone.0101765.s001.doc]

Table S1. Total number of mist-netted and banded adults for each species during the breeding season in 2010 at Forêt Montmorency (Québec, Canada).

| Species | Scientific name | Number of banded individuals |
| --- | --- | --- |
| Swainson's Thrush | *Catharus ustulatus* | 23 |
| Magnolia Warbler | *Setophaga magnolia* | 22 |
| White-throated Sparrow | *Zonotrichia albicollis* | 16 |
| American Redstart | *Setophaga ruticilla* | 11 |
| Yellow-rumped Warbler | *Setophaga coronata* | 11 |
| Ruby-crowned Kinglet | *Regulus calendula* | 8 |
| Golden-crowned Kinglet | *Regulus satrapa* | 7 |
| Red-breasted Nuthatch | *Sitta canadensis* | 6 |
| Gray Jay | *Perisoreus canadensis* | 5 |
| Black-throated Green Warbler | *Setophaga virens* | 4 |
| Blackpoll Warbler | *Setophaga striata* | 3 |
| Dark-eyed Junco | *Junco hyemalis* | 3 |
| Northern Waterthrush | *Parkesia noveboracensis* | 3 |
| Philadelphia Vireo | *Vireo philadelphicus* | 3 |
| American Robin | *Turdus migratorius* | 2 |
| Lincoln's Sparrow | *Melospiza lincolnii* | 2 |
| Black-capped Chickadee | *Poecile atricapillus* | 1 |
| Boreal Chickadee | *Poecile hudsonica* | 1 |
| Chipping Sparrow | *Spizella passerina* | 1 |
| Common Yellowthroat | *Geothlypis trichas* | 1 |
| Fox Sparrow | *Passerella iliaca* | 1 |
| Northern Parula | *Setophaga americana* | 1 |
| Ovenbird | *Seiurus aurocapilla* | 1 |
| Red-winged Blackbird | *Agelaius phoeniceus* | 1 |
| Swamp Sparrow | *Melospiza georgiana* | 1 |
| Total |  | 138 |
